# Whole Genome Sequencing Based Characterization of Extensively Drug-Resistant *Mycobacterium tuberculosis* Isolates from Pakistan

**DOI:** 10.1371/journal.pone.0117771

**Published:** 2015-02-26

**Authors:** Asho Ali, Zahra Hasan, Ruth McNerney, Kim Mallard, Grant Hill-Cawthorne, Francesc Coll, Mridul Nair, Arnab Pain, Taane G. Clark, Rumina Hasan

**Affiliations:** 1 Department of Pathology and Microbiology, Aga Khan University Hospital, Karachi, Pakistan; 2 School of Nursing and Midwifery, The Aga Khan University Hospital, Karachi, Pakistan; 3 Faculty of Infectious and Tropical Diseases, London School of Hygiene and Tropical Medicine, London, United Kingdom; 4 Pathogen Genomics Laboratory, King Abdullah University of Science and Technology (KAUST), Thuwal, Kingdom of Saudi Arabia; 5 Sydney Emerging Infections and Biosecurity Institute and School of Public Health, Sydney Medical School, University of Sydney, Sydney, Australia; St. Petersburg Pasteur Institute, RUSSIAN FEDERATION

## Abstract

Improved molecular diagnostic methods for detection drug resistance in *Mycobacterium tuberculosis* (MTB) strains are required. Resistance to first- and second- line anti-tuberculous drugs has been associated with single nucleotide polymorphisms (SNPs) in particular genes. However, these SNPs can vary between MTB lineages therefore local data is required to describe different strain populations. We used whole genome sequencing (WGS) to characterize 37 extensively drug-resistant (XDR) MTB isolates from Pakistan and investigated 40 genes associated with drug resistance. Rifampicin resistance was attributable to SNPs in the *rpo*B hot-spot region. Isoniazid resistance was most commonly associated with the *kat*G codon 315 (92%) mutation followed by *inh*A S94A (8%) however, one strain did not have SNPs in *kat*G, *inh*A or *oxy*R-*ahp*C. All strains were pyrazimamide resistant but only 43% had *pnc*A SNPs. Ethambutol resistant strains predominantly had *emb*B codon 306 (62%) mutations, but additional SNPs at *emb*B codons 406, 378 and 328 were also present. Fluoroquinolone resistance was associated with *gyr*A 91–94 codons in 81% of strains; four strains had only *gyr*B mutations, while others did not have SNPs in either *gyr*A or *gyr*B. Streptomycin resistant strains had mutations in ribosomal RNA genes; *rps*L codon 43 (42%); *rrs* 500 region (16%), and *gid*B (34%) while six strains did not have mutations in any of these genes. Amikacin/kanamycin/capreomycin resistance was associated with SNPs in *rrs* at nt1401 (78%) and nt1484 (3%), except in seven (19%) strains. We estimate that if only the common hot-spot region targets of current commercial assays were used, the concordance between phenotypic and genotypic testing for these XDR strains would vary between rifampicin (100%), isoniazid (92%), flouroquinolones (81%), aminoglycoside (78%) and ethambutol (62%); while *pnc*A sequencing would provide genotypic resistance in less than half the isolates. This work highlights the importance of expanded targets for drug resistance detection in MTB isolates.

## Introduction

*Mycobacterium tuberculosis* (MTB) caused 9 million cases of tuberculosis (TB) and 1.5 million death worldwide in 2013 [1]. Drug resistance in MTB increases the burden due to the disease and complicates both diagnosis and treatment. Multidrug-resistant (MDR)-TB is caused by MTB resistant to at least the two first line drugs i.e. rifampicin and isoniazid. Globally, in 2013, an estimated 480,000 people developed TB while, there were about 210,000 deaths due to TB [1]. Extensively drug resistant (XDR)-TB is caused by MDR-TB strains resistant to fluoroquinolone and to any one of the injectable aminoglycosides; amikacin, kanamycin or capreomycin. In Pakistan, MDR-TB comprises 4–5% of all TB cases, and XDR-TB is 4–5% of the MDR-TB cases [2,3]. Appropriate MDR-TB management requires drug susceptibility testing to guide treatment regimens. The usual turnaround time for phenotypic MTB drug susceptibility testing (DST) is about 8–10 weeks, often leading to delays in initiation of appropriate therapy. In high TB burden low resource settings facilities for mycobacterial culture and DST are limited. Therefore, rapid genotypic methods for detection of drug resistant MTB strains are required for more effective management of TB.

The Xpert MTB/RIF assay (Cepheid, USA) assay was rolled out at a global level since 2012; it detects rifampicin resistance by targeting the ribosomal polymerase *rpo*B gene [4]. The LiPA MTBDR*plus* (Hain Lifescience GmbH, Germany) assay targets *rpo*B for rifampicin resistance and *kat*G and *inh*A genes for isoniazid resistance. The LiPA MTBDR*sl* (Hain Lifescience GmbH, Germany) targets *gyr*A, *rrs* and *emb*B for fluoroquinolone, aminoglycoside and ethambutol resistance respectively [5]. Whilst these assays have significant value and allow rapid diagnosis of MDR-TB it is apparent that further improvements in molecular DSTs are required. MTB is largely clonal [6,7]. The TBDReaM database (http://www.tbdreamdb.com), catalogues greater than 1400 SNPs associated with drug resistance [8]. Studies of drug resistant MTB have shown that mutations in hot- spot regions, and those occurring beyond hypervariable regions vary according to both strain type and geographical location [9,10,11]. Thus, it is important for all possible resistance conferring mutations or single nucleotide polymorphisms (SNPs) related to each anti-tuberculous drug to be identified. Whole genome sequencing (WGS) facilitates the identification of SNPs, insertions and deletions in genomic DNA [12]. Thus, WGS of MTB can reveal new SNPs in genes previously associated with drug resistance and also identify outbreak strains [13].

By providing information on multiple genes simultaneously WGS facilitates the understanding of relationships between genes and their interactions such as in the case of fitness conferring compensatory mutations which occur due to the presence of drug induced selection pressure. Compensatory mutations are believed to play a role in conferring fitness to drug resistant MTB strains by affecting the growth rate of the isolates [14,15,16]. Compensatory mutations in *rpo*B genes which aid fitness of rifampicin resistant strains are well documented [17]. Knowledge of additional resistance mutation and compensatory mutations in MTB will be important for determining appropriate treatment for MTB strains. Here we have used WGS analysis to describe XDR-TB strains from Pakistan and identify SNPs in genes known to be associated with drug resistance to particular drugs.

## Materials and Methods

### Ethics statement

This study was approved by the Ethical Review Committee, The Aga Khan University.

### Strain selection

The *M*. *tuberculosis* strains studied were those collected from 2004–2009. Mycobacterial strains were obtained from the strain bank of Aga Khan University Clinical Microbiology laboratory. Demographic information on patients was obtained from clinical laboratory records and collected as part of good clinical practice. The hospital and its clinical laboratory are accredited by the Joint Commission of International Accreditation (JCIA, USA). MTB isolates were identified through a random selection method. Each strain had its unique identification number which at the time of testing was anonymised and unlinked with patient demographic information. The XDR-TB (n = 37) and drug susceptible (n = 5) isolates studied here were spoligotyped previously [3].

### Spoligotyping

Spoligotype patterns for each strain were obtained as described previously [18], and also confirmed *in silico* from the sequencing reads using *SpolPred* software [19].

### Culture and drug susceptibility testing

*M*. *tuberculosis* strains were isolated from Lowenstein-Jensen media and MGIT (Becton Dickinson, Franklin Lakes, NJ, and USA). *M*. *tuberculosis* was identified using BACTEC NAP TB differentiation test (Becton Dickinson), growth on para-nitrobenzoic acid containing media, nitrate reduction, and niacin accumulation [20]. DST of these isolates was performed using an agar proportion method on enriched Middlebrook 7H10 medium (BBL Microbiology Systems, Cockeysville, MD, USA) at the following concentrations: rifampicin 1 μg/mL, isoniazid 0.2 μg/mL, streptomycin 2 μg/mL, and ethambutol 5 μg/mL. Pyrazinamide sensitivity was determined by using BACTEC 7H12 medium, pH 6.0, at 100 μg/mL (BACTEC PZA test medium, Becton Dickinson). MDR TB strains were further tested with ciprofloxacin 2 μg/mL, capreomycin 10 μg/mL, amikacin 5μg/mL, kanamycin 6μg/mL and ethionamide 5 μg/mL. The reference strain *M*. *tuberculosis* H37Rv was used as a control with each susceptibility testing batch [21].

### DNA extraction and Whole Genome Sequencing of MTB isolates

DNA was extracted by the cetyl-trimethyl ammonium bromide (CTAB) method [22]. One microgram of DNA was used for sequencing. All samples underwent WGS with 76-base paired end fragment sizes, using Illumina paired end HiSeq2000 technology, and the raw sequence data is available in the European nucleotide archive (http://www.ebi.ac.uk/ena/data/view/PRJEB7798). For each sample *trimmomatic* software (http://www.usadellab.org/cms/?page=trimmomatic) was used to remove low quality reads and trim low-quality 3’ ends of reads. Nucleotide positions in the reads with a quality score lower than Q20 were removed. High quality reads were then mapped to the H37Rv reference genome (Genbank accession: AL123456.3) using *BWA-MEM* software (http://bio-bwa.sourceforge.net). SAMtools (http://samtools.sourceforge.net) and GATK (https://www.broadinstitute.org/gatk) were used to call single nucleotide polymorphisms (SNPs) and small indels. Variants (with quality at least Q30) were then selected as the intersection dataset between those obtained from both programs. Sample genotypes were called using the majority allele (minimum frequency 75%) in positions supported by at least 20-fold total coverage; otherwise they were classified as missing. Samples with a proportion of missing genotype calls greater than 15% were filtered out [21]. Similarly, we excluded positions in the genome with more than 15% missing genotypes across samples. Larger indels were called using a consensus from paired end mapping distance or split read approaches [23,24,25,26]. For more detail, the data processing pipeline has been described [27]. Spoligotypes were inferred from the sequencing reads *SpolPred* software [19]. Variation density maps were generated using Circos software (http://www.circos.com).

### Single nucleotide polymorphisms (SNPs)

The SNP data obtained was analysed according to known resistance conferring genes for each anti-tuberculous drug. For rifampicin resistance, *rpo*B, *rpo*A and *rpo*C were analysed. For isoniazid resistance, *kat*G, *inh*A,*oxy*R*-ahp*C, *fpb*C, Rv1592C, Rv1772, Rv2242, *fab*D, *kas*A, *acc*D, *oxy*R, *ndh*, *fad*E24, and *nat* were investigated. For pyrazinamide resistance, the *pnc*A and *rps*A genes were studied. For ethambutol resistance, *emb*C, *emb*A, *emb*B, *emb*R, *ini*A, *ini*B, *ini*C, Rv3124, *man*B, PPE49 *and rml*D genes were studied. For streptomycin resistance, *rps*L, *rrs and gid*B genes were investigated. For flouroquinolone resistance, *gyr*A *and gyr*B genes were studied. For aminoglycoside resistance, *rrs* and *tly*A, and for ethionamide resistance *eth*A *and fab*G1 genes were analysed. SNPs were divided into synonymous (sSNPs) and non-synonymous (ns-SNPs) mutations. Each SNP was compared with that listed in TBDReaMDB (http://www.tbdreamdb.com) database and other published reports of drug resistant MTB genotypes. For each strain, genotypic drug resistance findings were compared with phenotypic drug susceptibility results.

### Phylogenetic analysis

Phylogenetic analysis was performed in PHYLIP using a RAxML tree (Maximum likelihood phylogenetic software) with bootstrap to 1000 replicated [28]. Phylogenetic tree was visualized using Dendroscope software [29].

## Results

### Demographic information, phenotypic drug susceptibility and genotypic profile

The XDR strains comprised mainly CAS family isolates: CAS1-Delhi (n = 20), CAS (n = 2) and CAS2 (n = 1). Other strains belonged to EAT3-IND (n = 2), X3 (n = 1), T1 (n = 3) and Orphan (n = 8) lineages. Drug susceptible isolates were CAS1-Delhi (n = 2) and CAS (n = 3) strains.

The XDR strains were from the four provinces of Pakistan; Sindh (n = 21), Punjab (n = 16), Khyber Pakhtunkhwa Province (n = 4) and Baluchistan (n = 1), S1 Dataset. Sixty-two percent (n = 26) of MTB isolates were from males and 32% (n = 16) from females. The mean and median ages of patients was 34.2 and 31 years respectively (males: mean, 36.2, median, 34.5; female: mean 31.1, median, 26 years).

Isolates from Sindh comprised Central Asian Strain (CAS), T1, East African Indian (EAI), X and Orphan types and thus had more diverse lineages as compared to those from other provinces. However, within each province the CAS lineage was predominant, as described previously [30].

Shared type spoligotypes and unique MTB isolates were linked to their respective principal genetic group (PGG) based on the combinations of *kat*G*463-gyr*A*95* polymorphism [31], S1 Dataset and S2 Dataset. Most of the strain types (90.5%, 38/42) except, X3 (n = 1) and T1 (n = 3) belonged to the PGG1 lineage (based on *kat*G codon 463 CTG (Leu) and *gyr*A codon 95 ACC (Thr) polymorphisms). PGG1 strains included CAS, EAI and Orphan strains. One, X3 isolate was grouped in PGG2 as it showed *kat*G 463 CGG (Arg) and *gyr*A codon 95 ACC (Thr) polymorphism. Three T1 isolates with *kat*G 463 CGG (Arg) and *gyr*A codon 95 A*G*C (Ser) mutations were grouped in PGG3.

### Polymorphisms in genes associated with drug resistances

**Fig 1:**
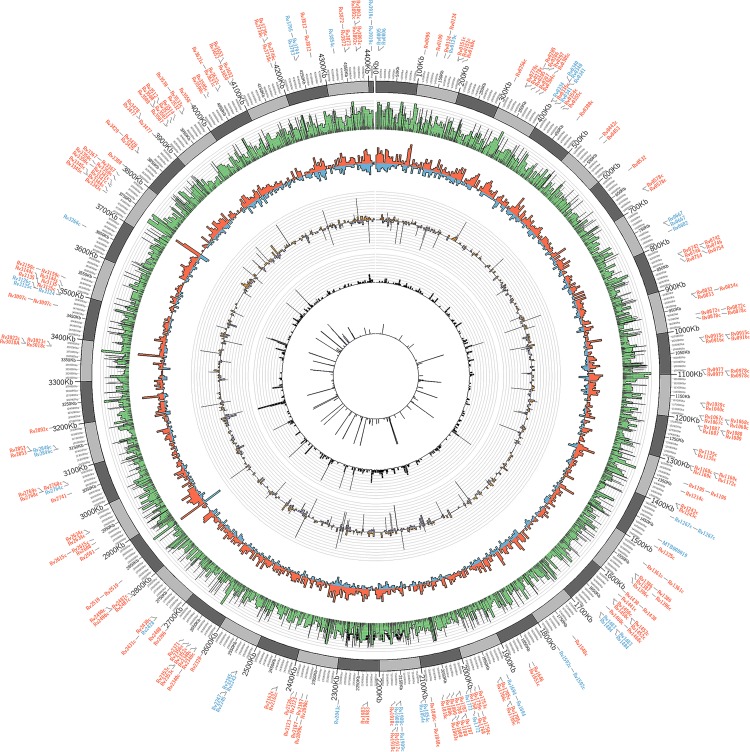
Comparative drug resistance genes information of *Mycobacterium tuberculosis* study isolates. Circos plot of *Mycobacterium tuberculosis* highlighting the genomic regions (Rv) where drug resistance associated SNPs were identified (colour coded in blue). The 5 inwards tracks represent SNP density, Non-synonymous/synonymous densities, small insertion/deletions densities, large deletion density and uniqueness track.

**Fig 2:**
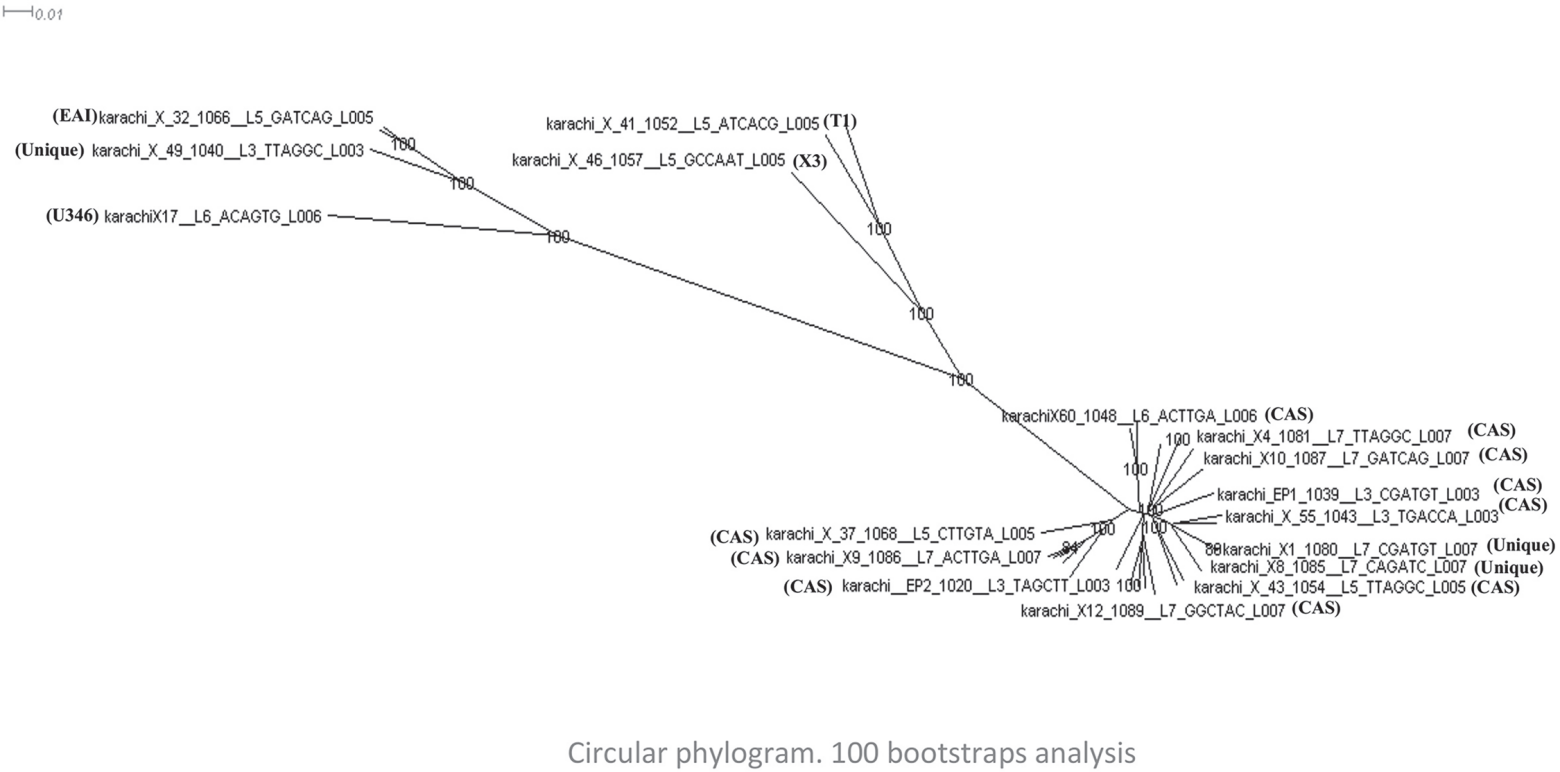
Circular phylogram based on the polymorphisms in the drug resistance genes of *Mycobacterium tuberculosis* isolates. Maximum likelihood analyses were performed with single nucleotide polymorphism identified in the drug resistance genes using Dendroscope software, version 3.2.8. Scale bar indicates 1% nucleotide divergence in the genes. Branch length corresponds to number of genetic polymorphisms inferred.

A visual description of SNPs densities throughout the genome of MTB isolates was compiled by a circos plot (Fig. 1). The maximum likelihood analysis is demonstrated by the circular phlyogenetic tree based on the polymorphism in the drug resistance genes using Dendroscope software version 3.2.8 [32], (Fig. 2). The clustering pattern of the strains demonstrated that T1 and X lineage strains clustered together (PGG3 and PGG2 groups respectively), and were distinct from CAS and EAI strains which clustered in a similar node. However, EAI remained distant from other PGG1 isolates in the phylogenetic tree.

**Fig 3:**
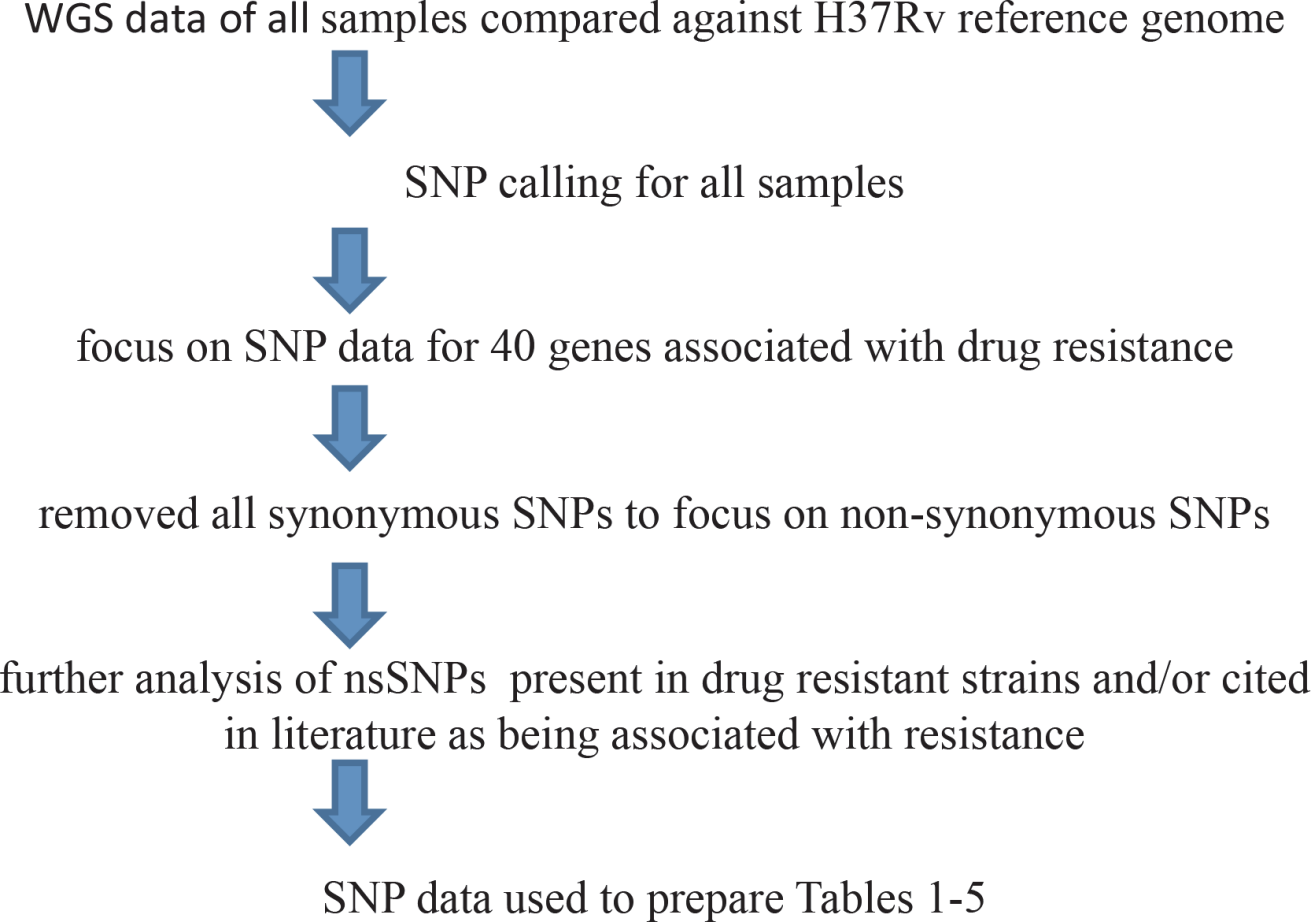
Process for identification of SNPs in genes associated with drug resistance. The flow diagram describes the manner in which SNPs were identified for inclusion in the prepared Tables.

By comparison with the H37Rv reference genome, SNPs were identified in 40 genes associated with drug resistance (Fig. 3). All synonymous SNPs (sSNPs) were removed from the analysis so that only non-synonymous SNPs (nsSNPs) are displayed in S2 Dataset. This nsSNP dataset is further described in Tables 1–5, which focus on the known resistance-determining regions in particular MTB genes.

**Table 1:** SNPs leading to rifampicin, isoniazid and pyrazinamide resistance in XDR strains.

Lineage of XDR isolates	*rpo*B^#^	RIF Compensatory mutations	*kat*G	INH compensatory mutations	*pnc*A	No.
CAS1-Delhi	S531L	*rpo*C G433S	S315T		P69G	1
CAS1-Delhi (1), Orphan (1)	S531L	*rpo*C G433S	S315T			2
CAS	S531L	*rpo*C P434L,		S94A		2
EAI3-IND	S531L	*rpo*C A172V, *rpo*C Q435A, *rpo*C P601L	S315T		I5T	1
CAS1-Delhi	S531L	*rpo*C V1252L	S315T		V41 stop	1
CAS1-Delhi	S531L	*rpo*C L516P	S315T			2
Orphan	S531L	*rpo*C A172V, *rpo*C G332R	S315T		P62S	2
CAS1-Delhi	S531L	*rpo*C L507V, *rpo*C R741C	S315T			1
CAS1-Delhi	S531L	*rpo*C V483G	S315T			1
X3	S531L	*rpo*C G594E	S315T		I6M	1
CAS1-Delhi	S531L	*rpo*C A492P	S315T			1
Orphan	S531L	*rpo*C A172V, *rpo*C P601L	S315T		M175T	1
CAS1-Delhi	S531L	*rpo*C V483A	S315T			2
CAS1-Delhi	S531L			S94A	D63H	1
T1	S531L	*rpo*A D324Y	S315T		P54L	2
CAS1-Delhi	S531L		S315T			1
CAS1-Delhi (1), T1 (1)	S531L	*rpo*C N416S	S315T			2
Orphan	M515I, D516Y		S315T		T76P	2
CAS1-Delhi	M515I, D516Y		S315T		Q141P	1
CAS1-Delhi (1), Orphan (1)	M515I, D516Y		S315T			2
CAS2	D516Y		S315T		V130M	1
CAS1-Delhi	D516Y		S315T			2
Cas1-Delhi (1), Orphan (1)	D516V		S315T			2
CAS1-Delhi	D516V		S315N			2
EAI3-IND	H526R	*rpo*C A172V, *rpo*C P601L	Y413H	*oxy*R g38a, *acc*D M439I, *acc*D A142V		1

All strain were resistant to rifampicin (RIF, 1 μg/ml), isoniazid (INH, 1 μg/ml) and pyrazinamide (PZA, 100μg/ml). ‘^#^’ mutations in the rifampicin- resistance determining region (RRDR), the number in parenthesis indicates the number of MTB isolates from lineage

**Table 2:** SNPs in genes conferring resistance ethambutol.

Lineage of XDR isolate	Phenotype*	*emb*B	No. of specimens
CAS1-Delhi (9), Orphan (3), T1 (1)	R	M306I	13
Cas1-Delhi (5), T1 (2)	R	M306V	7
Cas1-Delhi	R	M306L	1
EAI3-IND	R	M306V, E378A, G406A	2
CAS2	R	D354A, Q497P	1
X3	R	D328Y	1
Orphan	R	E378A	3
CAS1-Delhi	R	G406A	1
CAS	R	Q497K	2
Orphan	R	I72L #	1
CAS1-Delhi (3), Orphan (1)	R	D1024T #	4
CAS1-Delhi	R	wt #	1

‘*’ strains were tested against resistant to ethambutol (5 μg/ml),

‘#’ mutation outside the *emb*B ethambutol resistance determining region (ERDR), the number in parenthesis indicates the number of MTB isolates from each lineage

**Table 3:** Combination of SNPs in genes conferring resistance to streptomycin.

Lineage of XDR isolates	Phenotype*	*rps*L	*rrs* (500 region)	*gid*B	Number of specimens	Percentage of resistant isolates (n = 32)
CAS1-Delhi (5), T1 (3), Orphan (3)	R	K43R			11	34
CAS1-Delhi	R	K43R	A514C		1	3
Orphan	R		A514C		2	6
CAS1-Delhi	R		A514T		1	3
CAS1-Delhi	R	K43R	A514C	Q87 Stop	1	3
CAS1-Delhi	R		A906G	W148 Stop	1	3
CAS	R	K88R	A906G	P75S	1	3
CAS1-Delhi	R			G71 Stop	2	6
CAS (1), X3 (1)	R			P75S	2	6
CAS1-Delhi	R			W45 Stop	1	3
Orphan	R			A82P	1	3
CAS2	R			A119D	1	3
CAS1-Delhi (6), EAI3-IND (1)	R				6	19
CAS1-Delhi (2), Orphan (2), EAI3-IND (1)	S				5	

‘*’ strains resistant to streptomycin (STR, 2 μg/ml), the number in parenthesis indicates the number of MTB isolates from each lineage

**Table 4:** Combination of SNPs in genes conferring resistance to fluoroquinolone.

Lineage of XDR isolates	*gyr*A^#^	*gyr*B	Number of specimens
CAS1-Delhi (9), CAS2 (1)	D94G		10
Orphan	D94G	M291I	2
Orphan (5), CAS1-Delhi (3), T1 (1)	A90V		9
CAS1-Delhi (2), CAS (2)	D94Y		4
CAS1-Delhi	A90V, D94Y		1
CAS1-Delhi	S91P		1
T1	D94N		1
X3	D94Y	T500N	1
T1		S447F	1
EAI3-IND		M291I	2
Orphan		M291I, A432V	1
CAS1-Delhi			4

All strains were resistant to ciprofloxacin (2μg/ml) and ofloxacin (2 μg/ml). ‘^#^’ mutations in the *gyr*A quinolone- resistance determining region (QRDR), the number in parenthesis indicates the number of MTB isolates from each lineage

**Table 5:** SNPs present in XDR strains related to aminoglycoside resistance.

Phenotypic data*	Genotypes	*rrs* (1400 region)	Number of specimens
R to AMK, KAN, CAP	CAS1-Delhi (6), T1 (2), Orphan (2), X3 (1)	A1401G	11
	Orphan	G1484T	1
	EAI3-IND (1), CAS1-Delhi (1)	wt	2
R to AMK, KAN	CAS2(1), CAS1-Delhi (11), CAS (2), EAI3-IND (1), Orphan (3)	A1401G	18
	CAS1-Delhi (3), Orphan (1), T1(1)	wt	5

‘*’ resistance determined at amikacin (AMK, 6 μg/ml), kanamycin (KAN, 6 μg/ml) and capreomycin (CAP, 6 μg/ml), ‘wt’ wild type indicates absence of SNPs in the *rrs* 1400 gene, the number in parenthesis indicates the number of MTB isolates from each lineage

### Polymorphisms conferring rifampicin and isoniazid resistance

Resistance to rifampicin is associated with mutations in the RNA polymerase gene sub-unit B, *rpo*B. However, there is a fitness cost associated with the acquistion of *rpo*B mutation and rifampicin resistant MTB strains often acquire compensatory mutations in *rpo*C and *rpo*A genes to their improve growth [33]. Similarly, there is a fitness cost associated with acquisition of isoniazid resistance, and while *kat*G followed by the *inh*A gene mutations are most common, a number of other genes are thought to contribute to resistance [34]. We first evaluated SNPs in the context of MDR by studying the rifampicin-resistance determining region (RRDR) in *rpo*B, and compensatory mutations in *rpo*C and *rpo*A. For isoniazid resistance we analysed the *kat*G and *inh*A genes, and also investigated sequences of *oxy*R-*ahp*C, *fbp*C, Rv1592C, *ndh*, Rv2242, *fab*D, *acc*D, *fad* E24 and *nat*. Combinations of mutations observed in the XDR strains related to rifampicin and isoniazid are illustrated in Table 1. All the XDR isolates exhibited mutations in the RRDR of *rpo*B gene; codons 531 (n = 24), 516 (n = 12) and 526 (n = 1). The *rpo*B codon 531 mutation was present in PGG1, PGG2 and PGG3 isolates whereas, a mutation at codon 526 was observed in one PGG1 group EAI3-IND isolate. Mutations at codons 515 and 516 were present concurrently in each case, in CAS and Orphan strains.

Twenty-two XDR-TB isolates with compensatory mutations in *rpo*C and *rpo*A genes had the low fitness cost mutation *rpo*B S531L. One isolate with the *rpo*B H526R mutation had two *rpo*C SNPs. In all, we observed 15 different *rpo*C SNPs and one *rpo*A SNP. The most common was *rpo*C A172V which present in 5 isolates and *rpo*C P601L which was present in 3 isolates.

Polymorphisms at codon 463 of *kat*G were observed among all the PGG1 isolates. Resistance to isoniazid could be attributed to the *kat*G codon in 34/37 of cases and most strains (86%) had low fitness cost mutation, *kat*G S315T while, two strains had *kat*G S315N. Two strains had a *inh*A S94A mutation in the absence of *kat*G mutations. We did not observe any *inh*A promoter mutations amongst the XDR isolates studied. One EAI3-lineage strain had a *kat*G Y413H change.

In summary, if rifampcin and isoniazaid resistance were tested in these XDR strains using only MDR hot-spot region based targets (*rpo*B, *kat*G and -15 region *inh*A), 100% of rifampicin and 89% of isoniazid resistance would be detected.

When additional isoniazid resistance associated genes were analysed we found the *oxy*R g142a nucleotide substitution to be present in 4 strains together with a *kat*G S315T mutation (S2 Dataset). The EAI3 lineage strain (with *kat*G Y413H) had the mutations *oxy*R g38a, *acc*D M439I and A142V; in addition to *rpo*B H526R, and *rpo*C A172V and P601L mutations. We also observed additional SNPs present only in resistant strains with a *kat*G S315T mutation; Rv1592C (G9D, n = 4), *ndh* L104F and E360K, Rv2242 (A156V), *fab*D (A3T), *acc*D (E224D and A243V), *fad*E24 (Q85R), *nat* (T175A).

### Polymorphisms conferring resistance to pyrazinamide

All of the XDR strains were resistant to pyrazinamide treatment (Table 1). Non-synonymous SNPs in the *pnc*A gene were observed in 14 (38%) strains, whereby 11 different SNPs were observed (S2 Dataset). Twenty-three pyrazinamide resistant isolates did not reveal any nsSNP in *pnc*A; two such strains had the *rps*A mutation Q1228R.

Both pyrazinamide and isoniazid are pro-drugs which are converted to their active forms by enzymatic activity within the mycobacterium. We explore an association between isoniazid and pyrazinamide resistance by comparing *kat*G and *pnc*A mutations within strains. Of the XDR strains, thirteen had *pnc*A and *kat*G S315T mutations, while one strain with a *pnc*A mutation had *inh*A S94A (Table 1).

### Polymorphisms in the genes for ethambutol resistance

All of the XDR strains were resistant to ethambutol. The ethambutol-resistance determining region (ERDR) is primarily in the *emb*B gene and we found that 23 strains to have *emb*B codon 306 mutations, where M306I was most common (Table 2). Based on this data, commercial molecular diagnostic assays targeting *emb*B codon 306 would detect ethambutol resistance in 62% of the XDR strains studied here.

Other ethambutol resistant strains had SNPs at *emb*B codon 1024 (n = 4), codon 497 (n = 3), codon 406 (n = 1), codon 378 (n = 3), codon 328 (n = 1) and codon 72 (n = 1), S2 Dataset.

We analyzed additional genes associated with ethambutol resistance; *emb*C, *emb*A, Rv3124, *rml*D, *ini*A, *ini*B, *ini*C and PPE49. Different combinations of SNPs were observed in these ethambutol-resistance associated genes possibly, as compensatory mutations and or lineage associated mutations. Two EAI3-IND isolates (X5 and X32) had a distinct combination of mutations; *emb*B M306V, E378A and G406A, *emb*A P913S, *emb*C T270I, N394E, rmlD S257P and *ini*A H481Q (S2 Dataset). Two Orphan isolates (X17 and X57) also had a distinct combination of mutations; *emb*B E378A, *emb*A P913S, *emb*C T270I, N394E, rmlD S257P, *ini*A H481Q, *ini*C R481C and PPE49 M67L. Another isolate had a SNP in Rv3124 (V273I) (S2 Dataset) while one strain displayed a single nucleotide (T) deletion at codon 173 of *ini*A gene at 519 position.

### Polymorphisms conferring resistance to streptomycin

The mechanism of streptomycin resistance in the XDR strains was investigated by analyzing sequences of the ribosomal genes *rps*L, *rrs* (500 region) and *gid*B. The *rps*L mutation K43R was found in 41% of streptomycin resistant isolates; this SNP was found independently (n = 11) and also in combination with *rrs* A514C (n = 2), Table 3. The *rps*L mutation K88R was found in combination with *rrs* A906G and *gid*B P75S. Three strains had *rrs* A514C mutations only. There were eight different SNPs in *gid*B. Eight streptomycin resistant strains had *gid*B mutations only, without *rps*L and *rrs* (500 region) SNPs. However, six streptomycin resistant and five sensitive strains were without mutations in *rrs*, *rps*L *or gid*B (S2 Dataset).

### Polymorphisms in the genes for fluoroquinolone resistance

Mutations in *gyr*A and *gyr*B genes were analysed to investigate flouroquinolone resistance in the XDR strains. The most common mutation in the quinolone-resistance determining region (QRDR) of *gyr*A gene covering codons 90–98 was A94G, followed by A90V and A94Y (Table 4). All CAS family strains had *gyr*A QRDR mutations. One X3 strain had *gyr*A D94Y together with *gyr*B T500N. In total, 81% of isolates had SNPs in the QRDR of *gyr*A, which is the target region in currently available commercial diagnostic assays for flouroquinolone resistance.

Polymorphism at codon 95 of *gyr*A was observed among PGG1 and PGG2 isolates (S2 Dataset). The *gyr*A, G668D polymorphism was present in PGG3 (n = 3) isolates belonging to T1 lineage.

Of the eight strains which did not have *gyr*A QRDR SNPs, four had SNPs in the *gyr*B gene. One T1 strain only had the *gyr*B S447F mutation, two EAI3 strains only had the M291I mutation and an Orphan isolate had a gyrB M291I and A432V mutations. In one strain, a three nucleotide deletion was observed at codon 683 of *gyr*B gene at 7158 position. In all, four CAS1-Delhi XDR strains without *gyr*A or *gyr*B mutations.

### Polymorphisms conferring resistance to aminoglycosides

To investigate the mechanism for aminoglycoside resistance codon 1400 region of the *rrs* gene was analysed. Thirteen XDR strains were resistant to all three aminoglycosides (amikacin, kanamycin and capreomycin). Twenty-four XDR strains were resistant to amikacin and kanamycin but sensisitive to capreomycin. The most common *rrs* mutation across lineages was A1401G. Of strains resistant to amikacin, kanamycin and capreomysin, eleven had the SNP *rrs* A1401G and one had G1484T, whilst 2 strains had no mutation in the 1400 region of *rrs* (Table 5). Of the strains resistant to amikacin and kanamycin, eighteen had the SNP A1401G whilst five did not have a SNP in the *rrs* 1400 region. As current commercial diagnostic assays for aminoglycoside resistance target *rrs* nt 1401, 78% of resistance would be detected in these XDR isolates.

In all, 6 aminoglycoside resistant isolates did not reveal any polymorphism in hyper-variable 1400 region of *rrs* gene (S2 Dataset).

### Polymorphisms in the genes for ethionamide resistance

Five XDR isolates resistant to ethionamide but none showed any polymorphism in *eth*A gene, but one one ethionamide resistant isolate had the mutation *eth*A R239G which was also present in an ethionamide sensitive isolate (S2 Dataset).

## Discussion

Our results present the first WGS based characterization of XDR-TB strains from Pakistan. We focused on SNPs in genes known to confer resistance to first- and second-line drugs and present data relevant to molecular diagnostic assays for drug susceptibility testing.

Consistent with published reports all the rifampicin resistant MTB isolates in this study revealed at least one *rpo*B gene codon mutation i.e. *531*, *526*, *516* or *515*, in the RRDR region [35,36,37,38]. The most common mutation was the *rpo*B S531L, corroborating with previous reports [33]. In 22/23 isolates, compensatory mutations in *rpo*C and *rpo*A genes were coincident with the low fitness cost *rpo*B531 mutation, supporting previous reports which demonstrate increased fitness in rifampicin resistant isolates as a consequence of these compensatory mutations [39,40,41]. One EAI lineage isolate had the *rpo*B H526R mutation together with two *rpo*C mutations. As the *rpo*B codon 526 mutation has been shown to have a higher fitness cost than the codon 531 mutation [17] therefore, it would be an advantage for such an isolate to accumulate the *rpo*C compensatory mutations.

Based on the *kat*G*463-gyr*A*95* polymorphism 91% of the MTB isolates belonged to PGG1 of which the majority were CAS family isolates, as previously reported [30]. PGG1 is considered the oldest of MTB lineages and has been previously been reported from Asia [42]. The predominance of CAS strains suggests that they are well adapted to human populations in this region [43,44,45,46].

The most frequently observed isoniazid resistance associated mutation observed in our strains was *kat*G S315T, which has previously been shown to be associated with a growth advantage in strains [47]. Three isolates with *inh*A codon 94 mutations did not reveal *kat*G315 mutations, supporting a causal relationship of this mutation for isoniazid resistance [48,49].

Pyrazinamide is important as a first-line anti-tuberculous drug but phenotypic and genotypic discordance exists regarding testing its drug susceptibility. Consistent with previous reports, we found that more than 50% of resistant isolates did not reveal any mutation in *pnc*A gene [50]. Mutations in *pnc*A gene are thought to be the primary mechanism for pyrazinamide resistance, but our data consistent with others indicates that other resistance mechanisms may exist [51]. We found *rps*A mutations in two XDR isolates which did not have *pnc*A mutation, suggesting that this gene may play a role in resistance to pyrazinamide [52]. Additional pyrazinamide resistant strains had neither *pnc*A nor *rps*A mutations, and further supports the suggestion that mechanisms such an efflux pump for *pnc*A wild type resistant MTB isolates may be relevant [53].

The *emb*B306 SNP was found to be the most common mutation associated with ethambutol resistance in MTB strains, as reported previously [54]. The *emb*B306 mutation has been shown to be associated with high-level ethambutol resistance [55]. In addition, we also observed *emb*B codon 378, 406 and 497 mutations; which have reported previously [56] [55,57]. Our observations of distinct combinations of *emb*B and compensatory mutations in XDR isolates of EAI-IND and Orphan types suggest that some of these may lineage associated mutations. Especially, we observed a particular pattern of *emb*B, *emb*A, *emb*C, *rml*D and *ini* mutations was in four Orphan types, of which three were resistant and one was sensitive to ethambutol.

The most common streptomycin resistance associated mutation we observed was *rsp*L K43R. This mutation has been shown to play a role in reduced fitness cost and enhanced growth rate of resistant MTB isolates [47]. The K43R mutation has also been associated with high-level streptomycin resistance [58]. In the *rrs* gene we identified point mutations at positions 514 and 906, encoding for streptomycin resistance [58,59]. Previous studies have reported role of *gid*B polymorphism for low level of streptomycin resistance in MTB [60]. Here we found eight different *gid*B SNPs. Three strains had compensatory *gid*B SNPs with *rps*L and/or *rrs* mutations. Six streptomycin resistant isolates had *gid*B SNPs but did not have either *rps*L or *rrs* gene mutations. The occurrence of *gid*B mutations in strains reduces drug pressure for selection of highly resistant isolates, therefore it has been suggested that high-level streptomycin resistant strains (e.g. *rps*L mutants) arise more frequently in *gid*B mutants than in wild-type cells [61]. We observed six streptomycin resistant strains without mutations in *rps*L, *rrs* or *gid*B. The aminoglycoside antibiotics inhibit protein translation therefore it is likely that in additional mutations may be relevant for inactivating the effect of streptomycin in inhibiting tRNA translocation in mycobacteria.

In the strains resistant to amikacin, kanamycin and capreomycin we found 16S rRNA gene *rrs1401* and 1484 mutations to be the most common. These *rrs* mutations have previously been associated kanamycin and capreomycin [39,56,62]. In addition, it has been shown that the *tly*A and *rrs* gene mutations jointly play a role for capreomycin resistance [63]. However, we did not observe any *tly*A mutations amongst our strains.

We found that across MTB lineages, the most common fluoroquinolone resistance associated mutation was at *gyr*A codon 94, as shown previously [56,64]. Lineages other than CAS (X3, T1, EAI and Orphan) had *gyr*B SNPs in the 400 region. The *gyr*B gene has previously been shown to result in flouroquinolone resistance [65]. Four XDR strains did not have *gyr*A or *gyr*B mutations. This may be explained by the fact that additional mechanisms such as efflux pumps may be involved in a generating resistance flouroquinolone [66]. The predominant lineage amongst our XDR isolates was CAS and there is less representation of the other strain types. However, this data is suggestive of the association of flouroquinolone resistance in CAS lineages with *gyr*A QRDR region.

In the case of ethionamide, we found that none of the isolates had *eth*A SNPs which were particular to ethionamide resistance, as one isolate had *eth*A R239G mutation which was also present in an ethionamide sensitive isolate. A low frequency of mutation in *eth*A gene of ethambutol resistant isolates have also been reported earlier and may suggest existence of some other mechanisms such as efflux pump for ethionamide resistance [56,67].

Overall, the most frequently seen drug resistance mutations observed for isoniazid, rifampicin, streptomycin, ethambutol, aminoglycosides and ofloxacin were *kat*G*315*, *rpo*B*531*, *rps*L*43*, embB306, *rrs1401* and *gyr*A*94* respectively as previously reported [35,36,37,38,55,56,68,69]. Currently available commercial assays for detection of drug resistance (MTBDR*plus* and MTBDR*sl*) target the *rpo*B*531*, *516* and *526*, *kat*G*315*, *gyr*A*94*, *91* and *90* and *rrs1401* regions. Based on our data the frequency of drug resistance detection using such assays for these strains would have been 100% for rifampicin resistance, 92% for isoniazid resistance, 81% for fluoroquinolone resistance, 78% for aminoglycoside and 62% for ethambutol resistance. Our data indicates that SNPs in drug resistance genes vary with different MTB lineages. Therefore, it highlights the relevance for WGS data from prevalent global strain types in order to develop improved molecular diagnostic assays for resistance in MTB. Additional knowlegde of mutations that are compensatory to those that confer drug resistance will be important for prediction of levels of drug resistance and therefore, to guide treatment of drug resistance isolates.

## Supporting Information

S1 Dataset Drug susceptibility and demographic information of *Mycobacterium tuberculosis* isolates. (DOCX)

S2 Dataset An overview of SNPs observed in genes associated with drug resistance in XDR TB isolates. (XLSX)
