# Supplementary material for: Whole Genome Sequencing Based Characterization of Extensively Drug-Resistant Mycobacterium tuberculosis Isolates from Pakistan
Source: PLoS One. 2015 Feb 26;10(2):e0117771. doi: 10.1371/journal.pone.0117771 (PMC4342168; doi:10.1371/journal.pone.0117771)
Supplement: S1 Dataset — (DOCX) [file pone.0117771.s001.docx]

Supplementary Table 1. Drug susceptibility and demographic information of *Mycobacterium tuberculosis* isolates

| **Strain ID** | **Isolates Resistance to the Drugs^#^** | **Gender** | **Age** | **Geographical location** | **Spoligotype** | **PGG^** |
| --- | --- | --- | --- | --- | --- | --- |
| EP1 | Susceptible to all | M | 27 | Sind | CAS1-Delhi | PGG1 |
| EP2 | Susceptible to all | M | 5 | Sind | CAS1-Delhi | PGG1 |
| S4 | Susceptible to all | M | 20 | Sind | CAS | PGG1 |
| S5 | Susceptible to all | M | 70 | Sind | CAS | PGG1 |
| C3 | Susceptible to all | F | 36 | Sind | CAS | PGG1 |
| X1 | I R P S E F Cap K Ak | M | 19 | Sind | Orphan | PGG1 |
| X4 | I R P S E F K Ak | F | 28 | Punjab | CAS2 | PGG1 |
| X5 | I R P S E F K Ak | M | 34 | Sind | EAI3-IND | PGG1 |
| X6 | I R P S E F K Ak | F | 16 | Sind | CAS1-Delhi | PGG1 |
| X7 | I R P S E F K Ak | M | 63 | KPK | Orphan | PGG1 |
| X8 | I R P S E F K Ak | M | 40 | Sind | Orphan | PGG1 |
| X9 | I R P S E F K Ak | M | 70 | Punjab | CAS1-Delhi | PGG1 |
| X10 | I R P E F K Ak | M | 50 | Punjab | CAS1-Delhi | PGG1 |
| X11 | I R P S E F Cap K Ak | M | 34 | Punjab | CAS1-Delhi | PGG1 |
| X12 | I R P S E F K Ak | M | 28 | Punjab | CAS1-Delhi | PGG1 |
| X13 | I R P S E F K Ak | F | 25 | Sind | CAS1-Delhi | PGG1 |
| X14 | I R P E F K Ak | M | 50 | Punjab | CAS1-Delhi | PGG1 |
| X16 | I R P S E F K Ak | M | 50 | Sind | CAS1-Delhi | PGG1 |
| X17 | I R P S E F K Ak | F | 20 | Sind | Orphan | PGG1 |
| X18 | I R P S E F K Ak | M | 35 | KPK | CAS | PGG1 |
| X21 | I R P S E F K Ak | F | 32 | Punjab | CAS1-Delhi | PGG1 |
| X22 | I R P S E F K Ak Em | F | 55 | Punjab | CAS | PGG1 |
| X32 | I R P E F Cap K Ak Em | M | 45 | Sind | EAI3-IND | PGG1 |
| X33 | I R P S E F Cap K Ak | M | 16 | Sind | CAS1-Delhi | PGG1 |
| X37 | I R P S E F K Ak | M | 17 | KPK | CAS1-Delhi | PGG1 |
| X39 | I R P S E F Cap K Ak | M | 20 | Punjab | CAS1-Delhi | PGG1 |
| X40 | I R P S E F Cap K Ak | M | 18 | Sind | CAS1-Delhi | PGG1 |
| X41 | I R P S E F Cap K Ak Em* | F | 21 | Punjab | T1 | PGG3 |
| X42 | I R P S E F K Ak | F | 45 | Baluchistan | CAS1-Delhi | PGG1 |
| X43 | I R P S E F Cap K Ak | F | 55 | Punjab | CAS1-Delhi | PGG1 |
| X44 | I R P S E F Cap K Ak | M | 49 | Punjab | CAS1-Delhi | PGG1 |
| X45 | I R P F E Cap K Ak | M | 50 | Punjab | Orphan | PGG1 |
| X46 | I R P S E F Cap K Ak | M | 40 | Sind | X3 | PGG2 |
| X47 | I R P S E F Cap K Ak Em* | F | 25 | Punjab | CAS1-Delhi | PGG1 |
| X48 | I R P S E F K Ak | F | 19 | Sind | CAS1-Delhi | PGG1 |
| X49 | I R P F K Ak | F | 24 | Sind | Orphan | PGG1 |
| X55 | I R P S E F K Ak | M | 25 | KPK | CAS1-Delhi | PGG1 |
| X56 | I R P S E F K Ak Em | F | 20 | Punjab | T1 | PGG3 |
| X57 | I R P S E F K Ak | F | 27 | Sind | Orphan | PGG1 |
| X58 | I R P S E F Cap K Ak Em* | M | 30 | Sind | T1 | PGG3 |
| X60 | I R P S E F Cap K Ak | M | 35 | Sind | Orphan | PGG1 |
| X61 | I R P S E F K Ak | F | 50 | Punjab | CAS1-Delhi | PGG1 |

^# I=Isoniazid, R= Rifampicin, P= Pyrazinamide, S= Streptomycin, E= Ethambutol, F= Fluoroquinolones, Cap= Capreomycin, K= Kanamycin,^

^Ak= Amikacin, Em= Ethionamide; * Total Drug Resistant, ^PGG = Principal Genetic Group^
